# “Better be awake”—a role for awake extracorporeal membrane oxygenation in acute respiratory distress syndrome due to *Pneumocystis* pneumonia

**DOI:** 10.1186/s13054-019-2703-3

**Published:** 2019-12-23

**Authors:** Klaus Stahl, Benjamin Seeliger, Marius M. Hoeper, Sascha David

**Affiliations:** 1 Department of Gastroenterology, Hepatology and Endocrinology, Hannover Medical School, Hannover, Germany; 2 Department of Respiratory Medicine and German Centre of Lung Research (DZL), Hannover Medical School, Hannover, Germany; 3 Department of Nephrology and Hypertension, Hannover Medical School, Carl-Neuberg-Str.1, 30625 Hannover, Germany

To the editor

With interest we read the letter by Rilinger and coworkers who reported retrospective data on 18 patients with severe *Pneumocystis carinii* pneumonia (PcP)-associated adult respiratory distress syndrome (ARDS) supported with extracorporeal membrane oxygenation (ECMO) [1]. Overall hospital survival was 22% and with 50 vs. 8%, considerably more favorable in HIV than with other underlying immunosuppressive conditions. The authors concluded that ECMO support should mainly be considered for HIV-associated PcP.

Recently, our group has also reported the use of ECMO in this specific ARDS cohort [2]. The cohorts are strikingly similar in terms of group size (16 in our study), observation period (10 years vs. 8 years), relation of HIV to non-HIV patients, and most demographic characteristics including age, BMI, and importantly ARDS severity. However, the overall hospital survival rate was 31% in our series and we did not observe an inferior survival in non-HIV patients compared to HIV patients (30 vs 33%, *p* = 0.51, Fig. 1).

**Fig. 1:**
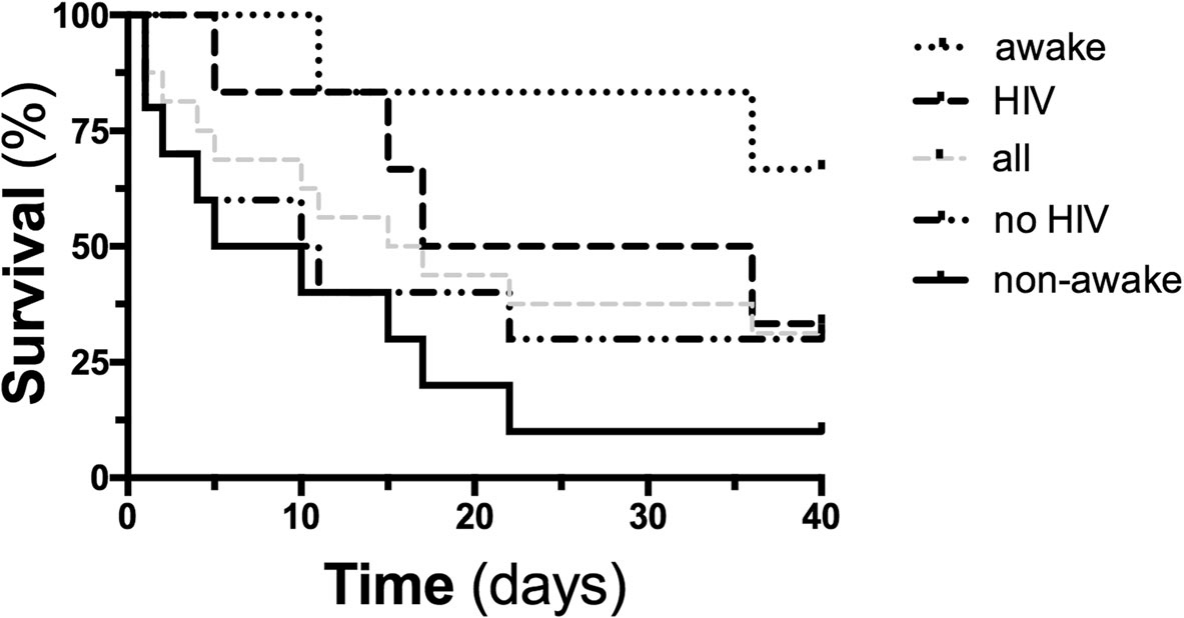
Survival in intubated vs. awake and in HIV vs. no HIV patients with PcP-associated ARDS receiving ECMO support*.* Kaplan-Meier graphs showing the 40-day survival course in awake (*n* = 6) and intubated (*n* = 10), HIV (n = 6) and no HIV (n = 10) patients, as well as all VV-ECMO patients with PcP-associated ARDS (mortality awake ECMO 2/6, 33% vs. intubated ECMO 9/10, 90%, *p* = 0.01, mortality HIV patients 4/6, 67% vs. no HIV patients 7/10, 70%, *p* = 0.51)

In contrast to Rilinger’s cohort, we employed a concept called “awake ECMO” in a subset of patients, who were conscious and spontaneously breathing during ECMO. Of note, 4 of the 6 patients, who primarily received awake ECMO support, survived until discharge from the hospital (67%) with better survival compared to primarily intubated ECMO patients (10%, *p* = 0.011). The awake ECMO strategy was a predictor for survival in our overall cohort of PcP patients (OR 18, 95% CI 1.2–260.9, *p* = 0.034), with a comparable proportion of HIV (2/6, 33%) and non-HIV patients (4/10, 40%). Despite the inherent limitations on non-controlled observations and small sample size, we think that the use of an awake ECMO concept may partially explain the better survival rate in our cohort.

Using awake ECMO might avoid complications associated with sedation and prolonged invasive mechanical ventilation such as pneumothorax, ventilator-associated pneumonia, ventilator-induced lung injury, systemic inflammation, and multi-organ damage [3, 4]. PcP usually leads to an isolated single organ failure without accompanying systemic complications such as septic shock, thus perhaps presenting an ideal scenario for consideration of an awake ECMO strategy [5]. Rilinger’s cohort appears comparable in this regard. Although not reporting on the necessity of hemodynamic support measures, a rather low degree of extra-pulmonary organ failure indicated by moderate SOFA scores and low proportions of renal replacement therapy were reported. We therefore believe that an awake ECMO strategy should be further explored in patients with PcP and ARDS.

## Data Availability

Not applicable.
